# Evaluating Healthy Corner Stores: A Survey of Assessment Tools Used in the San Francisco Bay Area, 2016

**DOI:** 10.5888/pcd14.170002

**Published:** 2017-06-01

**Authors:** Benjamin Chrisinger

**Affiliations:** 1 Stanford Prevention Research Center, Stanford University School of Medicine, Palo Alto, California

## Abstract

Stakeholders from healthy corner store programs in the San Francisco Bay Area convened in November 2015 to discuss the future of programmatic and collaborative efforts. This study’s objective, to gather and synthesize the types of evaluation tools used in the 9-county region, was identified as one of several priorities. Tools were collected via an online survey in July 2016, and data were extracted for comparison, including data on the number and types of food items, nutritional standards, and store characteristics. Twenty-five evaluation tools were collected, and differences were found in nutritional standards, terminology, and use of validated measures. Discrepancies between evaluation tools should be reconciled to make robust regional comparisons.

## Objective

Across the United States, programs have been implemented among corner store retailers to improve the availability of healthy food options, especially in low-income areas without access to larger food retailers (1). Although many initiatives are evaluated at the local level, various tools and methods are used, challenging efforts to consider regional effects or collaborations (2–5). Various corner store programs exist in the 9 San Francisco Bay Area counties (Alameda, Contra Costa, Marin, Napa, San Francisco, San Mateo, Santa Clara, Solano, and Sonoma). Our objective was to catalog and synthesize the types of information collected by evaluations of these programs.

## Methods

In November 2015, a meeting of program managers of Bay Area healthy corner stores and other stakeholders agreed on the need to understand when, where, and how evaluations are conducted. As an exploratory step, an online survey was developed and sent to all 77 attendees in July 2016 to collect and classify the types of evaluations being used. Respondents were encouraged to upload all relevant evaluation tools with their completed online study survey. In addition to uploading the evaluation tool, each respondent was asked to provide information on when the tool was used, the scope of the tool, whether it was from a standardized source and externally validated or developed by the local stakeholder, kinds of information collected (eg, prices, availability), and method of data collection (eg, interview, paper/tablet-based audit). Responses and evaluation tools were accepted through August 2016.

Survey responses were collected and organized. Several types of data were extracted from the evaluation tools. These included data on food items and nutritional standards and store characteristics, such as the number of registers in the store and whether the store participated in federal food assistance programs. Evaluation tools were classified into 3 categories according to the method used: in-store observation or audit, consumer interview, and owner or manager interview. Evaluation tools were also coded by the number of items assessed in 7 food categories (dairy, protein, grain, fruit and vegetable, snack, beverage, and other food), nonfood goods or services sold, and store characteristics. All categories were divided into subcategories; for example, dairy was subcategorized by milk (eg, 1%, skim, whole, flavored/unflavored), milk alternatives (eg, soy beverages, almond milk), and other dairy items (eg, yogurt, cheese) (Box). Descriptive statistics were generated for type of method used, category, and subcategory. Results were also presented to a group of healthy food retail stakeholders in the San Francisco area for additional refining in December 2016.

**Box. Examples of Terminology Used in San Francisco Bay Area Healthy Corner Store Evaluation Tools by Categories and Subcategories of Food and Beverage Items, 2016**

**Table:** 

Category /Subcategory	Examples of Terminology
**Dairy**	
Milk	Low-fat or skim milk, 1% milk, 2% milk, whole milk, flavored, nonflavored, no sugar added
Milk alternatives	Milk alternatives, lactose-free/nondairy beverage, soy beverage, soy products, almond milk, flavored, nonflavored, no sugar added
Other	Yogurt, cheese, butter, ice cream
**Protein**	
Meat	Red meat, ground meat, chicken, poultry, fish, other meat
Other protein	Nut butter, eggs, legumes, tofu
**Grain**	
Cereal	Cereal
Whole grains	Dried whole grain, whole grains, dry foods
Pasta/noodles	Pasta, whole-grain pasta or noodles
Bread	Bread, whole-wheat bread, 100% whole-grain bread, white bread, gluten-free bread, nonflour tortilla, tortilla corn, tortilla wheat, tortilla flour, pita bread, sourdough bread, pan dulce, bagels, fresh French bread
**Fruits and vegetables**	
Vegetables	Canned vegetables, canned vegetables or soup, fresh vegetables, frozen vegetables
Fruits and vegetables	Frozen fruits and vegetables, fresh fruits and vegetables, dried and canned fruits and vegetables; produce
Fruits	Dried fruit, fruit cups, fresh fruit, canned fruit
**Snack food**	
Snacks	Healthy snacks, snacks, healthy snacks/baked goods salty, healthy snacks/baked goods sweet, salty snacks
Bars	Energy and power bars, granola and cereal bars, granola bars, cereal bars
Nuts/seeds	Nuts, nuts/seeds, nuts seeds and trail mix no candy
Other snacks	Granola; trail mix with candy, hummus packs, popcorn, rice cakes
Jerky	Dried meat/jerky, beef jerky
Candy	Gum and mints, candy, candy/gumballs
Desserts	Sweet desserts, cookies and cakes, cookies
Chips/pretzels	Pretzels, chips and pretzels, chips
**Beverages**	
Water	Water/seltzer, flavored water, water
Juice	100% fruit juice, juices
Nonsugar sweetened	Coconut water, unsweetened beverages, unsweetened tea, diet/noncaloric beverages, coffee
Sugar sweetened	Sports drink, energy drink, sweetened beverages, sugar-sweetened beverages, soda
Alcohol	Alcohol
**Other foods**	
Frozen meals	Frozen healthy meals, frozen meals
Other foods	Baby food, smoothies, staple groceries, prepared foods, canned products
**Nonfood goods/services**	
Tobacco/e-cigarettes	Tobacco, flavored tobacco, e-cigarettes
Other services	Check cashing, telephone cards, ATM, lottery tickets
**Store characteristics**	
Food-related	Product quality, product placement, product pricing, local foods, culturally acceptable foods, checkout area,
Marketing-related	Product promotion, health promotion, advertising
Store environment	Cleanliness, aesthetics, number of registers
Financial accessibility	Accept SNAP, accept WIC, accept credit cards
Community issues	Hiring practices, languages spoken, living wage, community participation, wheelchair-accessibility

## Results

Twenty-five unique evaluation tools were reported via online survey (n = 14) or email to the author (n = 11). Of these, 5 were excluded because they did not provide sufficient information to extract summary data for comparison. Only 1 evaluation tool was externally validated (4), whereas others were created or compiled by local researchers or managers. Among types of methods, in-store observations or audits were most prevalent (n = 11), followed by consumer interviews (n = 5), and owner/manager interviews (n = 4). On average, by type of method used, in-store observations or audits had 25.7 items (standard deviation [SD], 14.4), consumer interviews had 24.6 items (SD, 13.3), and owner/manager interviews had 12.5 items (SD, 13.3).

**Figure 1:**
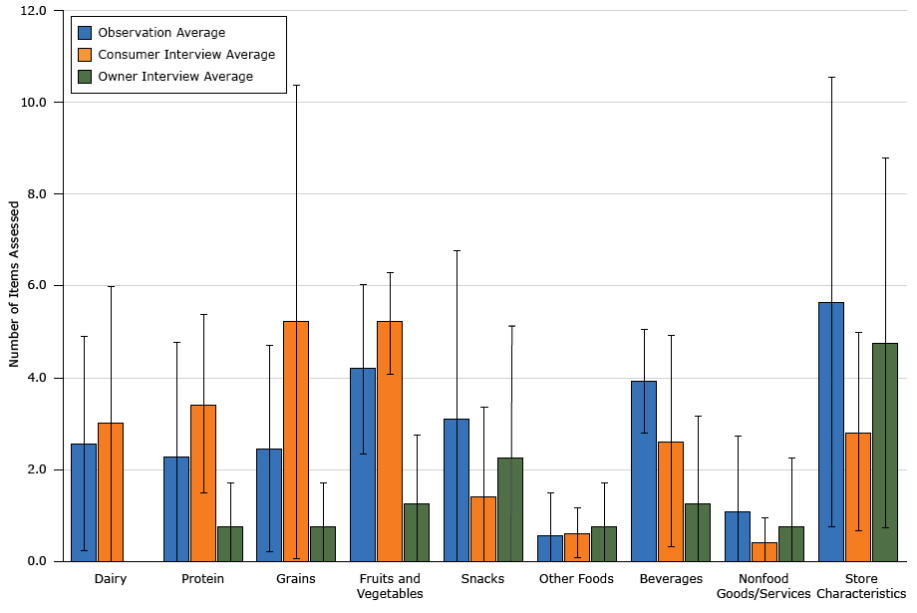
Average number of items assessed in evaluations of healthy corner stores, by type of method used, San Francisco Bay Area, 2016. None of the owner interviews assessed dairy, so no bar appears for that item. Error bars indicate standard deviation. **Table:** 

Evaluation tools collected information on an average of 22.8 items (SD, 14.2), with fruits and vegetables, beverage, and store characteristic categories having the highest average number of items (Figure 1). Of the 20 evaluation tools, 18 included beverage items and/or fruits and vegetables and 16 included some type of assessment of store characteristics.

Variable	Observation, Average No. (SD)	Consumer Interview, Average No. (SD)	Owner Interview, Average No. (SD)
Dairy	2.5 (2.3)	3.0 (3.0)	0.0 (0.0)
Protein	2.3 (2.5)	3.4 (1.9)	0.8 (1.0)
Grain	2.5 (2.3)	5.2 (5.2)	0.8 (1.0)
Fruits and vegetables	4.2 (1.8)	5.2 (1.1)	1.3 (1.5)
Snacks	3.1 (3.7)	1.4 (1.9)	2.3 (2.9)
Beverages	3.9 (1.1)	2.6 (2.3)	1.3 (1.9)
Other food	0.5 (0.9)	0.6 (0.5)	0.8 (1.0)
Nonfood goods and/or services	1.1 (1.6)	0.4 (0.5)	0.8 (1.5)
Store characteristic	5.6 (4.9)	2.8 (2.2)	4.8 (4.0)

Nutritional language or standards varied by subcategory of food (Table). For example, one tool applied standards for sugar content in yogurt (≤13 g sugar for children’s yogurt, ≤20 g sugar for others), while others only recorded its presence or absence. Disagreement between nutrition claims was not widespread, though exceptions existed; for example, grain and snack food categories included multiple definitions of allowable sugar content. When presented to the stakeholder group, many of these findings appeared to be consistent with practitioner observations and generated questions for future research.

**Table:** Nutritional Vocabulary Used in Healthy Corner Store Evaluation Tools in the 9-County San Francisco Bay Area Region As of December 2016^a^

Product Category/Subcategory		Nutritional Language or Standard
**Dairy**	Milk	•Reduced fat •2% •1% •Low-fat or skim •Skim •No added sugar •Unflavored
	Milk alternative^b^	•No added sugar/sweetener •Unflavored
	Cheese^c^	•Skim •Low-fat •1%
	Yogurt^c^	•Low fat •Children’s: ≤13 g sugar/serving •Non-children’s: ≤20 g sugar/serving
**Protein**	Meat	•≤15% fat •Fresh •Skinless
	Nut butter^c^	•Unsweetened •No added oils •Nonhydrogenated
**Grain**	Cereal	•First ingredient whole grain, <9 g sugar/serving •Whole grain, <7g sugar •<7g sugar or >10% daily value of fiber •≥3g fiber, ≤12 g sugar
	Bread	•≥10% daily value of fiber, whole grain first ingredient •Whole wheat •100% whole grain •Gluten-free
**Fruits and vegetables**	Vegetable, canned	•<290 mg sodium/serving •≤140 mg sodium •In water •No added fat, sugar, sweetener
	Vegetable, fresh	Does not count potatoes or onions; ≥1 dark leafy green (does not count iceberg lettuce)
	Fruits and vegetables, frozen	•No added fat, sugar, or sweetener •No added sauce/sugar
	Fruit, canned	•100% juice, no added sugar/syrup •Nonsyrup •No sugar added, no syrup/sauce
	Fruit, fresh	Does not count lemons and limes
**Snack food**	Snacks	•≤230 mg sodium; ≤13 g sugar •Fruit or vegetable based, healthy protein based, whole grain, dairy based; local •Whole grain (≥2 g fiber) •<10 g sugar, <10% daily value of fat
	Bars	•Whole grain, ≥2g fiber, ≤1g saturated fat, ≤14 g sugar •Energy bars (≤14 g sugar)
	Nuts and seeds	No added sugar, not honey-roasted
	Popcorn^c^	Low-fat/no butter
	Chips and pretzels	Baked
**Beverages**	Water	•Calorie-free flavored, plain, mineral, or seltzer •Carbonated (no sugar), plain (unflavored)
	Juice	•100% fruit juice •Unsweetened
	Non-sugar–sweetened	•No added sugar •0 g sugar
**Other food**	Frozen meals	<800 mg sodium, <8 g fat
	Prepared foods^c^	Healthy sandwiches

a Some of the nutritional vocabulary used in this table is nonstandard and reflects wording used in a healthy corner store evaluation or locality.

b Milk alternatives, such as soy and almond milk.

c Subcategory amended to identify object of nutritional language or standard.

## Discussion

Study results clarify the role of various evaluation tools for various purposes: establishing objective baseline conditions, documenting changes, and ensuring compliance with program goals (observational tools); understanding individual and community-level perceptions and attitudes about foods and retailers and gathering insights about neighborhood needs (customer interviews); and characterizing the uptake and sustainability of in-store interventions (owner interviews). Validated observational measures were reported by 3 counties (eg, the California Department of Public Health’s CX^3^ tool [4]), and standardized, nonvalidated instruments were used by 5 counties (eg, Center for Science in the Public Interest’s healthy checkout audit [6]). This study’s findings suggest that greater standardization and documentation of rigorous methods could be useful but would entail additional costs (eg, staff time, training).

To compare results and assess impact at a regional level, disagreements within item categories and between evaluation classes must be addressed. Across the 3 types of evaluation tools, the number of items varied most (SD >3.0) for store characteristics, grains, and snacks. Regional stakeholders have already initiated an effort to codify nutritional standards with a focus on snacks. This study provides additional motivation for similar work and identifies other possible priority areas for standardization.

This study has several limitations. Although efforts were made to reach all area stakeholders, some evaluation tools may have been excluded. Generalizability may also be limited to areas where political or logistical realities are amenable to similar stakeholder engagement.

To understand and compare standards in various methods of evaluating healthy corner stores in the 9-county Bay Area, it was necessary to take stock of existing evaluation tools. This study identified 3 general classes of evaluation tools and the categories and subcategories of items these tools recorded. This information may be useful in future collaboration or pooling of data. Insights from this study provide additional motivation for coordination in establishing regional standards.
